# Chronochemistry in neurodegeneration

**DOI:** 10.3389/fnmol.2014.00020

**Published:** 2014-03-31

**Authors:** Annalisa Pastore, Salvatore Adinolfi

**Affiliations:** Department of Clinical Neuroscience, Institute of Psychiatry, King’s College LondonLondon, UK

**Keywords:** Alzheimer’s disease, determinism, disease development, Friedreich’s ataxia

## Abstract

The problem of distinguishing causes from effects is not a trivial one, as illustrated by the science fiction writer Isaac Asimov in a novel dedicated to an imaginary compound with surprising “chronochemistry” properties. The problem is particularly important when trying to establish the etiology of diseases. Here, we discuss how the problem reflects on our understanding of disease using two specific examples: Alzheimer’s disease (AD) and Friedreich’s ataxia (FRDA). We show how the fibrillar aggregates observed in AD were first denied any interest, then to assume a central focus, and to finally recess to be considered the dead-end point of the aggregation pathway. This current view is that the soluble aggregates formed along the aggregation pathway rather than the mature amyliod fiber are the causes of disease, Similarly, we illustrate how the identification of causes and and effects have been important in the study of FRDA. This disease has alternatively been considered as the consequence of oxidative stress, iron precipitation or reduction of iron–sulfur cluster protein context. We illustrate how new tools have recently been established which allow us to follow the development of the disease. We hope that this review may inspire similar studies in other scientific disciplines.

## INTRODUCTION

### THE IMPACT OF DETERMINISM IN OUR LIVES

By philosophical choice or from our simple and most direct perception of reality, we are accustomed to consider the world as deterministic, i.e., as being ruled and regulated by the principle of causality. This principle states that each action or event is the consequence of a previous action or event. We cannot have the effect before the cause. We have plenty of everyday examples: being hit by a car while crossing a road implies that a car was driving along the road and run over a person who was crossing the road. Surely we could not have a car accident before the car’s arrival. Likewise, if a cake comes out from the oven this necessarily means that someone must have baked it and could not be the other way around.

Occasionally, some dreamers have considered the possibility that determinism could be violated and explored how this possibility could reflect on our life. A paradigmatic example is that of the scientist and science fiction writer Isaac Asimov, who wrote a spoof of the deterministic concept in a novel dedicated to thiotimoline (Asimov, 1948). This is supposed to be a wonder substance derived from the bark of the (fictitious) shrub Rosacea karlsbadensis rufo. Thiotimoline has the property of solubilising exactly 1.2 s (not a second before or after) before the researcher adds water to the compound, thus challenging the principle of cause and effect as in this case the effect would precede the cause… The author’s explanation for such extraordinary behavior is chemically “rigorous” although highly fictional: in the thiotimoline molecule, there is at least one carbon atom that has two of the four chemical bonds in the normal space and time dimensions; one of the other two bonds projects into the future and the other into the past, thus bridging time. As imagined by Asimov, if true, the consequences would be unimaginable: the substance could be used to create fantastic mechanisms that could be exploited in “chronochemistry,” a discipline of Asimov’s invention which only Russian scientists excel in while the Americans, more suspicious, are unfortunately left behind… 

Letting aside this and similar fictional possibilities, the concepts of determinism and causality remain simple and undeniable certainties that rule our everyday lives.

Where things go fuzzier is when we do not know the events that well. Not that we would doubt their deterministic nature but there might be cases in which it is difficult to distinguish between cause and effect. The concept acquires a dramatic importance when referred to medicine, a field where the problem of identifying the correct causality is directly related to diagnostics. Failure to identify the cause, or confusion between cause and effect, can direct the cure along false paths making it ineffective, if not dangerous. A heavy headache and stomach sickness could well be explained by assuming that having developing a headache we started also having an upset stomach. But it is equally possible that because of feeling sick we developed headache or even that the two events are uncorrelated and that the two pathological conditions came out at the same time, perhaps as the effect of a third event such as a viral attack or any other direct or indirect cause.

There are in principle three criteria that need to be met before we can say that there is a causal relationship between two events. First, we must be able to show that there is a temporal sequence of events and that the cause happened before the effect. Second, there should be co-variations of the cause and the effect: if one parameter influencing one event was increased, the other event should be affected in a simple binary relationship. Finally, it should be possible to rule out plausible alternative explanations that could explain the co-detection of the two events. Most often, establishing these three criteria is far from being easy, especially when we wish to apply them to biology or medicine.

In this review, we discuss the difficulties of identifying cause and effects in specific biological events that are directly related to neurodegeneration and the problem of identifying disease etiology. We show how the problem can be clarified by the possibility of identifying a clear “time zero” (i.e., the time point when the chain of events causing the disease starts) and by checking how the symptoms accumulate from this point. We shall consider two paradigmatic examples: the case of the amyloid-related Alzheimer’s disease (AD) and the example of Friedreich’s ataxia (FRDA), two diseases studied widely in the last few years. We shall also discuss the implications that these specific examples have for our understanding of the biological phenomena causing disease.

### THE PROBLEM OF ESTABLISHING CAUSES AND EFFECTS IN DISEASE ETIOLOGY

Alzheimer’s disease is an aging-related disease of increasing impact for the world population which involves impairment of memory, reasoning, abstraction, and language (Christensen et al., 2009). The cytological and biochemical complexity of this disease has made it difficult to find an easy agreement on the temporal sequence of events that lead to AD and, as a consequence, on the steps most amenable to drug treatment. AD was first diagnosed and studied by the Bavarian psychiatrist Alois Alzheimer, who identified the presence of unusual formations in the post-mortem brain of a 51 year old patient (Alzheimer, 1907). These lesions are known as amyloid plaques as amyloid plaques and neurofibrillar tangles according to their morphology and location (Selkoe, 2011; Serrano-Pozo et al., 2011). Only much later, it became clear that the lesions are proteinaceous formations that mainly contain, respectively, Aβ peptides that are degradation products of the “amyloid precursor protein” (or APP) (Kang et al., 1987) and the microtubule-associated protein (MAP) tau protein (Brion et al., 1985; Masters and Selkoe, 2012).

We now know that these two molecules are intrinsically unstructured polypeptides that are highly hydrophobic and have a high tendency to misfold and form fibrillar aggregates termed amyloids from their tintorial properties. It is accepted that amyloid fibers adopt a β-rich structure of the same type as that observed in silk (Marshall and Serpell, 2009). The conversion of α-helix or random coil conformations within normally soluble proteins into β-sheet rich assemblies is a common theme recurrent in several neurodegenerative diseases and has been shown to be a universal solution of the energy landscape common to most if not all proteins (Eichner and Radford, 2011; Ma and Nussinov, 2012).

Interestingly, the presence of plaques and tangles was interpreted differently through time. When Glenner and Wong (1984) first identified Aβ from post-mortem meninges of AD patients, the authors suggested that these formations could “provide a diagnostic test for AD and a means to understand its pathogenesis.” Other scientists argued instead that the plaques and tangles might “only” be end-stage lesions and that would provide little useful information about etiology and early pathogenesis. Nevertheless, plaques and tangles have been used as diagnostic tools for several decades. As a consequence many people adopted what is known as the “amyloid hypothesis,” which assumes that extracellular deposits of Aβ peptides are the fundamental cause of AD (Hardy and Allsop, 1991; Mudher and Lovestone, 2002; Luheshi et al., 2008). Inspired by these studies, amyloid fibers have been thoroughly studied and large attention has been paid to them not only in the AD field but in several other, otherwise uncorrelated diseases also associated to protein aggregates (Luheshi et al., 2008).

Association does not however, necessarily imply a causal relationship even though this inference is rather tempting. More recently, it has become progressively clear that AD could not after all be caused by the final mature amyloid fibers but by other species which form along the aggregation pathway. It has been suggested that the formation of amyloid fibers could even be a defense mechanism of the cell to protect itself from other more toxic species which have recently been identified in the so called oligomers (Hardy and Selkoe, 2002). These are assembly species formed along the aggregation pathway but well before the mature fibers are formed. The importance of the oligomers rather than mature fibers is supported by several lines of evidence.

Some patients develop severe symptoms well before plaques are formed or at least are detectable. Conversely, plaques can be found in the cortex of aged subjects who are at least apparently healthy, although it is possible that they might have not been tested for subtle cognitive dysfunctions. Oligomers, that is all forms that remain soluble after high-speed ultracentrifugation of brain extracts and not of fibrillar forms, are detectable in buffer-soluble extracts of post-mortem AD patients’ cortex (Shankar et al., 2008). These species are cytotoxic as shown for the first time by Lambert et al. (1998). Later on, injection of oligomeric (dimers) species into the cerebral ventricles of healthy adult rats confirmed that they transiently impair the memory of a learned behavior (Shankar et al., 2008). Aβ oligomers have been shown to cause neuronal death in culture and to block long-term synaptic depression (Lambert et al., 1998; Stine et al., 2003; Wang et al., 2004). There is a statistical correlation between cortical levels of soluble Aβ and the extent of synaptic loss and severity of cognitive symptoms (Kuo et al., 1996; Lue et al., 1999; McLean et al., 1999; Wang et al., 1999). Oligomers of the size of Aβ dimers but not monomeric species from the same brains were shown to facilitate long-term synaptic depression in the hippocampus and to decrease dendritic spine density (Shankar et al., 2008; Li et al., 2009b).

**FIGURE 1:**
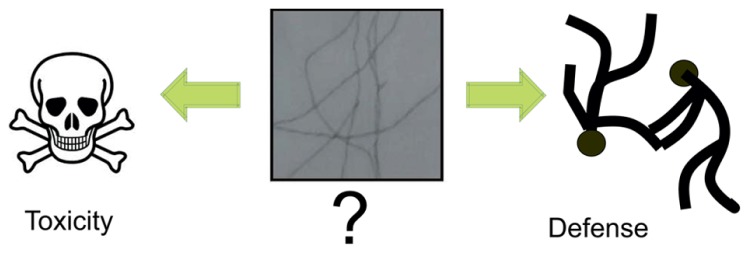
**Debated role of fibrillar amyloids.** A recurrent question which seems to become clear only recently is whether the fibrillar amyloid species observed in patients’ brains are the toxic species or rather a defense mechanism of the cell. The current view is that the toxic species are the soluble aggregates formed through the aggregation process. As such, the mature fibrillar fibres are the final point of this process.

Taken together, these findings support the hypothesis that small, soluble oligomers of human Aβ peptides are sufficient to induce several features of the AD phenotype, including synaptic loss, tau hyper-phosphorylation, neurofibrillary degeneration, and memory impairment, even in the absence of amyloid plaques. When we determine the presence of plaques, in fact, most of the damages have already taken place. Thus the fibrillar aggregates could be the ultimate effect, the escape and not the cause of AD (**Figure 1**).

The lesson we may learn from this story could be a hard one. It could be argued that confusion between cause and effect has been negative and has distracted researchers from the “real thing,” encouraging them to follow the wrong pathway. At least 10 years have been dedicated to a thorough study of the morphological, mechanical and structural properties of amyloids. However, we can convincingly argue back that it has not been a waste of time in the broader perspective of our fundamental knowledge of reality. It is now clear that amyloids are not “just” a rare or totally unwanted structure. Amyloid peptides are routinely found in hormones (Maji et al., 2009) and are produced by *E. coli* and other gram-negative enteric bacteria (Wang et al., 2008) and by the gram-positive bacterium *S. coelicolor *(Capstick et al., 2011). While some of the interest in amyloid fibers could be considered ineffective and dangerously delaying an anti-Alzheimer treatment, our “detour” into the amyloid world will certainly prove highly beneficial for many other areas of Biology in the close future.

### THE IMPORTANCE OF DETECTING THE VERY EARLY EVENTS OF A DISEASE

Let’s now consider the example of FRDA. This is a neurodegenerative disease caused by an abnormal expansion of a non-coding GAA triplet repeat in the first intron of the FRDA gene (Pastore and Puccio, 2013). This event causes lower expression of the FRDA gene product, the protein frataxin, through hetero-chromatization of the locus. The age of disease onset correlates inversely with the number of repeats. FRDA provides a unique example (of still ongoing research) in which causes and effects can easily be mixed up until the “‘right” experiments are designed to establish a temporal relationship between observations.

A large body of work has been produced since the discovery of the gene responsible for this ataxia (Campuzano et al., 1996). We now know that frataxin is a nuclearly encoded protein, produced in the cytoplasm and then imported into and matured in the mitochondrion where its primary function resides. To reach this conclusion it was noticed early on that only eukaryotic frataxins contained an N-terminal extension that could represent an import signal (Gibson et al., 1996). It was also noticed that the distribution of the frataxin gene in different organisms is fully consistent with the fusion event that leads to the formation of mitochondria. The hypothesis was soon after independently proven experimentally (Campuzano et al., 1997).

But what is the role of frataxin in mitochondria? One of the early observations was that of iron deposits in heart tissues of FRDA patients (Sanchez-Casis et al., 1977). This was confirmed by a deletion mutant in yeast (ΔYFH1) that showed a ten-fold increase in the iron concentration in mitochondria and an increase of hypersensitivity to oxidative stress (Babcock et al., 1997). A more complete investigation of the molecular defects present in patients carried out by Rustin and his group identified a deficient activity of iron–sulfur (FeS) cluster-containing subunits of mitochondrial respiratory complexes I, II, III and aconitase in endomyocardial biopsies (Roting et al., 1997). The authors made an attempt to create a temporal connection between the observed strong and yet very different phenotypes. They concluded that iron overload is the “causa prima” that generates oxidative stress through iron-catalyzed Fenton chemistry. This would in turn give rise to oxygen free radicals and iron precipitates. The latter are well known to target efficiently FeS proteins causing them to quickly lose their activities as observed in patients and yeast-null mutants generating the pathology. The authors did not, however, provide any indication of where iron could come to the equation which left the explanation somewhat unsatisfactory.

To address the question in a different way, several laboratories developed animal and cellular models that could allow the study of the multiple effects generated by the reduced production of frataxin as observed in disease. These studies rely on the high conservation of the frataxin gene along evolution, which makes possible to model the effects of frataxin deletion in different systems and compare the effects to those observed in patients’ cells. A homozygous mouse model in which the frataxin gene was drastically switched off was the first attempt to understand the primary events caused by the absence of the protein and the cellular phenotype of FRDA (Cossée et al., 2000). This homozygous deletion caused embryonic lethality a few days after implantation, indicating that frataxin is an essential protein in mammals. Surprisingly, though, no iron overload or iron precipitates were detected in degenerating embryos, in open contrast with the hypothesis that iron precipitates are the primary cause of the pathology, thus suggesting that they could be instead a side-effect. These findings were explained assuming that yeast frataxin could have a ferritin-like function as an iron storage chaperone within the mitochondria (Isaya et al., 2000). Cell death in mouse embryos would be the consequence of the lack of a scavenger rather than the result of iron precipitation, reinforcing the idea that iron deposits are a secondary event.

It is important however, to consider that the discrepancy between the data in yeast, in patients and in transgenic mice could be more simply explained by the unfair comparison of conditions detected at very different time points of the disease course. Iron precipitation is the most striking phenomenon observed in patients’ tissues, where the disease is usually present for years, and in yeast-null mutants grown for many generations before been used in experiments. In embryos instead, iron accumulation could hardly have time to build up, thus giving a better description of what the cause and the effects may be. Embryos could in facts be an ideal system to capture the early events close to what we shall call “time zero,” that is the disease onset.

To better understand the time relationship between events, different laboratories developed conditional knock-out mice for the frataxin gene (Puccio et al., 2001; Martelli et al., 2012) or yeast models in which the gene was put under a galactose-regulated promoter in yeast (Muhlenkhoff et al., 2002). Both systems would allow switching off the gene at a given time point so that this could be taken as “time zero” from which the effects could be followed with controlled molecular and functional assays. To obtain the same effect in *Drosophila*, an RNAi-mediated-suppression strategy was used (Anderson et al., 2005; Llorens et al., 2007). These models all revealed that frataxin deficiency primarily leads to strong defects in FeS cluster containing proteins and FeS cluster maturation followed by accumulation of iron deposits. In yeast, a defect in respiratory efficiency was also detected (Muhlenkhoff et al., 2002), following the disruption of FeS cluster maturation. In mouse, the FeS cluster deficit is observed before the first evidence of a cardiac dysfunction (Martelli et al., 2012). Interestingly, no evidence of oxidative damage was observed in this and in a *Drosophila* model (Anderson et al., 2005; Llorens et al., 2007). This observation led to the still open question of whether formation of oxygen free radicals (ROS) is at all essential to generate the cellular phenotype associated with FRDA.

The experiments described above were excellent attempts to generate animal models in which frataxin production could be controlled and lowered “to induce the disease,” starting the observation of the molecular and cellular phenotype at a well-defined time. However, although conceptually correct, such studies were often carried out without any attempt to investigate the actual time course of events: the phenotype was tested only at few time points which were often spaced different weeks apart. Unfortunately, as in AD, when the phenotype becomes observable most of the events that lead to it have already taken place.

A more direct approach became possible when the proteins associated with the FeS cluster biogenesis machine were purified, both in eukaryotes and prokaryotes (Adinolfi et al., 2009; Prischi et al., 2010). This allowed the possibility of reproducing the FeS cluster biogenesis machine *in vitro* and of studying the effect of frataxin on the formation of *de novo* synthesized FeS clusters without the complexity of the *in vivo/in* *cell* environments. What makes these experiments particularly attractive is also that the time course of the events can be followed directly and in a continuous way. Data are now available for the bacterial (Adinolfi et al., 2009; Prischi et al., 2010), yeast (Li et al., 2009a), and human (Tsai and Barondeau, 2010; Bridwell-Rabb et al., 2012) systems and have provided a great deal of new information. These studies clearly showed that frataxin and its orthologs play a critical role in regulating FeS biogenesis. These findings were rationalized by the report that frataxin and its orthologues bind specifically and stoichiometrically to the complex Nfs1/Isu (or IscS/IscU in bacteria; Tsai and Barondeau, 2010). These proteins are the two central components of the FeS cluster assembly machine, being respectively the desulphurase that provides sulfur for cluster assembly and the scaffold protein on which the cluster forms.

Much as this approach clarified some issues, it could however, be noticed that the *in vivo* and *in vitro* experiments so far described have a completely different time frame for the appearance of the effect of frataxin deficiency letting us wonder whether or how these results can really be compared to each other and reconciled.

To fill the gap and be able to observe and quantify the effects of frataxin in a time frame as close as possible to the *in vitro* studies, Tamarit and co-workers have recently suggested an alternative approach: they developed a yeast model placing frataxin under a controlled promoter (Moreno-Cermeno et al., 2010). The authors used tetO-YFH1 mutants where the addition of doxycycline to the growth medium allows an efficient and specific repression of frataxin expression. This drug is known not to affect other gene expression. The system was used to follow the effects of frataxin deletion *in vivo*, starting the measurements of different parameters at the time of addition of doxycycline, and following the time evolution using time-points close to the *in vitro* experiments (Moreno-Cermeno et al., 2010). Tamarit and co-workers’ results indicate that the deregulation of iron metabolism is the primary effect of frataxin depletion, followed by iron accumulation (visible after 4 h) that occurs at the early stages. They also demonstrated that increased oxidative damage and decreased concentration of FeS cluster proteins such as aconitase, are late defects visible only after 24 h, and might be considered as secondary effects that are a consequence of iron overload. A possible criticism of this study could be that the residual activity of the FeS cluster proteins after frataxin depletion could be explained by a longer half-life of these proteins. This possibility can be ruled out, since reduction of expression of proteins responsible for FeS cluster biogenesis leads to a more than 80% reduction of aconitase activity (Rodriguez-Manzaneque et al., 2002; Kumanovics et al., 2008). The presence of normal aconitase activity for 24 h after frataxin depletion cannot thus be attributed to the initial holo-aconitase pool, but indicates that the FeS cluster machine remains active even in the absence of frataxin.

**FIGURE 2:**
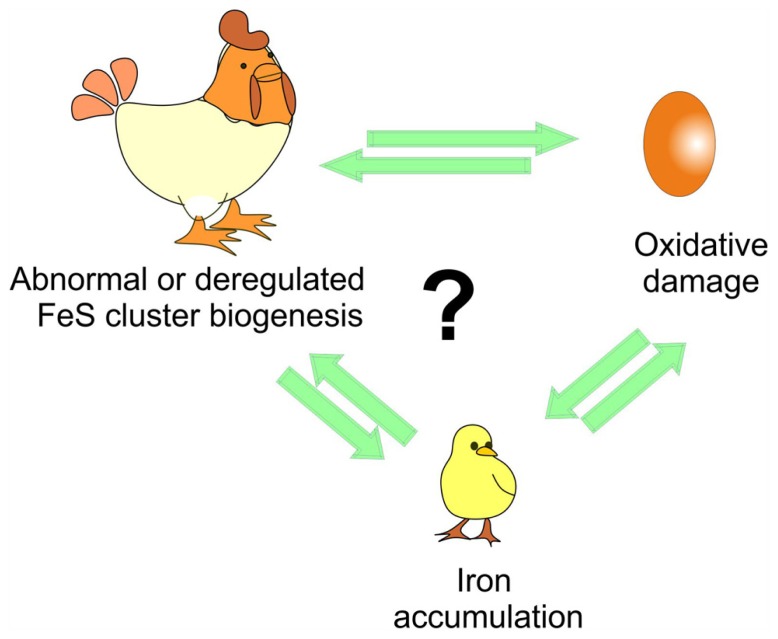
**Understanding the time course of the events triggered in disease.** The debate is whether iron accumulation is the cause or just an effect of iron–sulfur (FeS) cluster deregulation and/or of the oxidative stress and of course which of the two comes first as in the chicken and egg causality dilemma.

While more work is still needed to clarify several aspects, the approach proposed by Tamarit together with the *in vitro* studies provide powerful tools to clarify the temporal pipeline of FRDA and hold a tangible promise for understanding the cellular role of frataxin and its connection to disease (**Figure 2**).

## CONCLUSION

In this review we have discussed the difficulties of understanding disease etiology, a problem that, if anything, is becoming even more important in our molecular medicine era. The problem is of particular interest in neuroscience where many diseases are poorly understood and interconnected with several different and apparently uncorrelated symptoms. We discussed two examples in which causes and effects have been confused, AD and the less well known FRDA. The former exemplifies well how the same symptomatology can be considered the aetiological cause or even a defense mechanism which, rather than harming the organism, is activated to prevent further damages. The same concept has been transferred to other neurodegenerative diseases which share with AD the presence of protein aggregates. FRDA provides a unique example of how experiments can and must be tailored to clarify the time sequence of events. Although still ongoing, the research in this field has made enormous progresses from its early days and holds the promise of unveiling soon the exact mechanism of this still incurable disease.

The take home message that this discussion suggests is that much care needs to be taken before jumping to conclusions and assuming that all factors associated with a disease are its very cause. At the same time, we propose the importance of designing suitable experiments that may clarify the temporal relationship between events as the only way to understand the mechanisms responsible for disease onset.

## Conflict of Interest Statement

The authors declare that the research was conducted in the absence of any commercial or financial relationships that could be construed as a potential conflict of interest.
